# Elevated high sensitive C-reactive protein in fibromyalgia

**DOI:** 10.3389/fpsyt.2023.1237518

**Published:** 2023-11-30

**Authors:** Eva Beiner, Sergio Brenner Miguel, Hans-Christoph Friederich, Jonas Tesarz, PerPAIN Consortium

**Affiliations:** ^1^Department of General Internal Medicine and Psychosomatics, Medical Hospital, University of Heidelberg, Heidelberg, Germany; ^2^Institute for Applied Mathematics and Interdisciplinary Center of Scientific Computing (IWR), University of Heidelberg, Heidelberg, Germany

**Keywords:** fibromyalgia, hsCRP, inflammation, FMS, C-reactive protein

## Abstract

**Introduction:**

Fibromyalgia syndrome (FMS) is a complex chronic pain condition characterized by widespread pain and tenderness, fatigue, and sleep disturbances. Currently, factors contributing to FMS are considered to be multifactorial, and the involvement of inflammatory processes is a question of debate.

**Objective:**

The aims of this study were (1) to assess whether serum concentrations of high-sensitivity C-reactive protein (hsCRP) differ between individuals diagnosed with FMS and pain-free controls, (2) to determine whether these differences are associated with clinical symptoms, and (3) to explore whether the observed differences can be explained by specific covariates such as age, weight, and smoking status.

**Methods:**

An ANOVA was applied to identify differences of hsCRP levels between FMS and pain-free controls and an analysis of covariance (ANCOVA) was performed to investigate the dependencies of hsCRP with respect to covariates. To assess the reliability of our findings, we also utilized a Bayesian robust estimation model to determine the level of confidence associated with our results.

**Results:**

The results showed that individuals with FMS had higher hsCRP levels compared to healthy controls [*F*(1,106) = 8.802, *p* < 0.001] and that higher hsCRP levels were significant correlated with a higher symptom burden (*r* = 0. 287, *p* = 0.008) and more tender points (*r* = 0.307, *p* = 0.005). Further, hsCRP levels were significantly associated with weight (η^2^ = 0.154, *p* < 0.001), but independent of age (η^2^ = 0.005, *p* = 0.42), smoking status (η^2^ = 0.002, *p* = 0.623), or gender (η^2^ = 0.0045, *p* = 0.437), which resulted in an insignificant group effect between FMS and controls (η^2^ = 0.029, *p* = 0.052), even after controlling for covariates.

**Conclusion:**

In conclusion, this study provides evidence that sub-inflammatory processes correlate with clinical symptoms, which can be partly attributed to differences in weight, but cannot be fully explained by them. Further research is needed to elucidate the mechanisms underlying the association between hsCRP and FMS and to explore the potential therapeutic implications of targeting hsCRP in the management of FMS.

## 1 Introduction

The Fibromyalgia Syndrome (FMS) is a widely distributed disease that affects 2–7% of the population (1). Those patients are suffering from widespread pain, fatigue, sleep disturbances, concentration problems, and a high burden of somatic symptoms. Allodynia and hyperalgesia are also features of FMS, manifested as painful tender points distributed throughout the body (2). Overall, the symptomatology is very diverse, and a multifactorial etiology is assumed (3).

A lot of research has been done to investigate the pathophysiology of FMS, but still, this disease is not completely understood yet. Several factors seem to increase the risk to develop FMS, such as adverse experiences in the childhood or persistent stress, but also for example elevated body mass index (BMI), obesity, smoking, or other existing medical illnesses (4–6). Due to a high involvement of stress in the pathogenesis of FMS, biomarkers have become a focus of research. These biomarkers include stress associated markers as for example cortisol, catecholamines as (nor-)epinephrine, adrenocorticotrophin releasing hormone (ACTH), and corticotropin releasing hormone (CRH), but also inflammatory immune cytokines such as IL-6 or IL-8 and c-reactive protein (CRP) (7–9). Until today, the evidence of the involvement of those markers in the pathogenesis and development of FMS is not clarified.

The current evidence is heterogenous, some studies show elevated marker concentrations in individuals with FMS and some studies appear to find the opposite. Due to the involvement of some of those markers in the inflammation processes of the body and in pain processes, a relation between FMS and its symptoms with pro-inflammatory cytokines has been suggested, as these markers may contribute to the development of symptoms such as central pain sensitization (9–11). One such marker is C-reactive protein (CRP), which is commonly used as an indicator of acute inflammatory processes (12). CRP has come under increasing scrutiny in recent years, as studies have repeatedly reported elevated CRP levels in fibromyalgia syndrome. However, the literature is contradictory (13, 14). In a recent meta-analysis (8), the marker CRP was investigated in individuals with FMS compared to pain-free controls. Although there was a significant effect for elevated CRP in FMS, only a few studies were included in this meta-analysis with five studies showing elevated CRP concentrations in FMS (11, 15–18), whereas the other studies showed no effects of CRP between the groups (19–21). One possible reason for the conflicting results could be vague inclusion criteria that did not adequately exclude inflammatory comorbidities. In addition, imprecise analytical methods, and the lack of consideration of possible proinflammatory factors such as obesity or smoking should be taken into account. The introduction of highly sensitive CRP assays (hsCRP) now makes it possible to detect even subtle inflammatory activity (9), including subclinical processes (22), which may shed light on the underlying mechanisms of fibromyalgia. This now makes it possible to overcome possible biases in previous studies and to re-examine the significance of hsCRP in patients with FMS. Due to the heterogenous evidence (8, 13, 23), the aims of this study were (1) to assess whether serum concentrations of high-sensitivity C-reactive protein (hsCRP) differ between individuals diagnosed with fibromyalgia syndrome (FMS) and pain-free controls, (2) to determine whether these differences are associated with clinical symptoms, and (3) to explore whether the observed differences can be explained by the covariates age, weight, and smoking.

## 2 Materials and methods

### 2.1 Sample

The sample of the present study included participants that were part of a larger study for multilevel phenotyping of pain patients and identification of subgroups, for improving outcomes in chronic pain patients, through a personalized therapy approach (PerPAIN) (24), funded by the German Federal Ministry of Education and Research (BMBF). The focus of this study is an exploratory analysis based on the first data release from the ongoing recruitment phase of the PerPAIN cohort. The main objectives of the PerPAIN study are to expand a cohort of patients with musculoskeletal pain to identify specific subgroups and to conduct a feasibility study of a personalized treatment allocation algorithm [for more details, see Beiner et al. (24)]. The sample size for PerPAIN was calculated to achieve statistical power for these specific aims, rather than for comparisons between the FMS group and controls. At the time of our analysis, the PerPAIN trial was still actively recruiting participants. Therefore, the sample used in the present analysis represents only a preliminary subset of the final cohort planned for the PerPAIN trial. The aim of the first data release was to provide an initial description of the demographic and biomarker characteristics of this preliminary sample. Given this exploratory intent, we did not formally calculate the sample size for the comparison between FMS patients and controls. The study protocol was approved by the Ethics Research Committee II of the Faculty of Medicine, University of Heidelberg (2020-579 N) and will be carried out in compliance with the Helsinki Declaration. For further details on the study design see Beiner et al. (24).

### 2.2 Eligibility criteria

From 244 included participants, 214 participants were suffering from chronic pain, from which 125 were diagnosed with FMS and 30 were pain-free healthy controls. To be considered eligible for this study, individuals were required to fulfill the ACR 2016 criteria for FMS diagnosis (WPI ≥ 7, SSS ≥ 5 or WPI 4–6 and SSS ≥ 9) (25), and the validity of the diagnosis was verified by a trained physician through a standardized clinical assessment. Inclusion criteria were, next to the presence of FMS according the ACR 2016 criteria, age of at least 18 years, and ability to give informed consent. Individuals with inflammatory comorbidities (e.g., rheumatoid arthritis, psoriatic arthritis, systemic lupus, active tumor disease…), severe physical or severe psychological comorbidities, neurological disorders or pregnancy were excluded from the study. In addition, all individuals who were currently taking, or had previously taken, any anti-inflammatory medication [cortisone (*n* = 3), biologics (*n* = 10)] were excluded to avoid any confounding with the outcome variable. Pain-free controls were recruited by opportunity sampling and were subjected to the same inclusion criteria, plus the absence of any chronic pain condition. With the exclusion of three patients taking cortisone and 10 patients with a history of Biologica intake, finally, 80 individuals with FMS and 30 controls met the eligibility criteria and were included into analyses.

### 2.3 Procedure

Written consent was provided from all participants before enrollment. All participants underwent a standardized physical examination by a trained study physician. The FMS group underwent an even more detailed physical and clinical examination regarding the American College of Rheumatology (ACR) diagnostic criteria for FMS to validate the clinical diagnosis (25–27). Testing took place in a quiet room. Before starting the tests, the participants rested for half an hour in their respective environments. During this time, a medical history was taken and information about pain and functional disability was assessed. Blood markers were extracted before any study assessments took place.

### 2.4 Measures

The blood samples were analyzed in the central laboratory of the University Hospital Heidelberg. Standard operating procedures, according to the instructions of the manufacturer, were used. The blood samples were centrifuged for 10 min at 3.500 g. If the analysis was not directly carried out, the blood samples were stored at 4–8°. Levels of hsCRP was analyzed on a Dade Behring BN II (nephelometer analyzer) with a nephelometric assay (reagent kit OQIY 13).

The BMI was calculated from the height and weight measured on the day of the physical examination. The standard formula was used. The spatial extent of pain was measured using the widespread pain index (WPI) (26). The WPI consists of 19 body regions, each of which is evaluated for pain in the past week. The WPI score is calculated as the sum of all reported painful body regions and can range from 0 to 19, with higher scores indicating a greater extent of widespread pain. Generalized sensitivity to pressure stimuli was assessed semi quantitatively by a standardized manual probe (≈10 N/cm^2^ per second up to ≈ 40 N/cm^2^ max) by trained medical personnel at 18 tender points according to the ACR 1990 criteria (27). Tender points were counted with a total score from 0 and 18 with higher scores indicating higher myofascial tenderness. Somatic symptom burden was measured using the Somatic Symptom Scale (SSS-8) (28, 29) The SSS-8 consists of eight items that cover a range of physical symptoms commonly associated with various medical conditions. These symptoms include pain, fatigue, gastrointestinal distress, and cardiopulmonary symptoms. The questionnaire uses a five-point Likert scale, where respondents rate the frequency and severity of each symptom from “0” (not at all) to “4” (very severe).

### 2.5 Statistical analysis

In a first step, an ANOVA analysis was performed to identify whether hsCRP concentrations differ between individuals with FMS and pain-free controls. For the exploration of the associations between hsCRP and clinical symptoms, spearman correlation tests were used, with a Holm type correction to correct the *p* values. To further characterize the dependency between hsCRP, with respect to the two groups (FMS and pain-free controls) and covariates, an ANCOVA model was performed. To gain a more comprehensive understanding of the data and its validity, we decided to use Bayesian analysis to complement the frequentist approach and provide additional insight into the likelihood of different outcomes (30, 31). The robust Bayesian estimation uses a t-distribution and an unequal variance model, using the brms-function in R (32). For the Bayesian approach normal prior in the intercept and *b*-values, as well as a Cauchy prior on the sigma and an exponential prior on nu, were considered. Both, the ANCOVA as well as the Bayesian Model allowed the same interpretation, which is discussed in the results section. Before analysis, hsCRP values were log-transformed. All analyses were performed with R (Version 4.0.2).

## 3 Results

The mean age of the FMS group was 48.7 (±13.3) and 44.0 (±15.7) for the control group. The FMS group consisted of 71 women (88.75%), whereas the control group consisted of 12 women (40%). For further information, see Table 1. For the first research question, the ANOVA for differences of hsCRP between FMS and pain-free controls revealed a significant difference between the groups with *F*(1,106) = 8.802, *p* < 0.001, partial η^2^ = 0.77. With a mean of 2.71 (3.43) for the FMS group and 1.75 (3.88) for the control group, the FMS group showed higher hsCRP values compared to controls. The performed Bayesian approach led to an estimated effect of +0.84 with 95% credibility set [0.31, 1.37], which was consistent with the results of the ANOVA. Here, a normal prior in the intercept and *b*-values, as well as a Cauchy prior on the sigma and an exponential prior on nu, were considered.

**Table 1:** Descriptive information.

	FMS (*n* = 80)	HC (*n* = 30)
Age, M (SD)	48.7 (13.3)	44.0 (15.7)
Females, % (*n*)	88.75% (71)	40% (12)
BMI (kg/m^2^), M (SD)	27.42 (6.57)	24.79 (3.94)
Spatial extent of pain (WPI 0–19), M (SD)	11.9 (3.05)	0.5 (1.11)
Somatic symptom burden (SSS-8), M (SD)	7.99 (2.04)	1.07 (1.44)
Tender points, M (SD)	11.24 (5.02)	0.23 (0.83)
Smokers, % (*n*)	16.25% (13)	10.00% (3)
hsCRP, M (SD)	2.71 (3.43)	1.75 (3.88)

*n*, Sample size; M, Mean value; SD, Standard deviation; Min, Minimum; Max, Maximum; BMI, Body mass index; WPI, Widespread pain index; and SSS-8, Somatic symptom scale.

**Figure 1:**
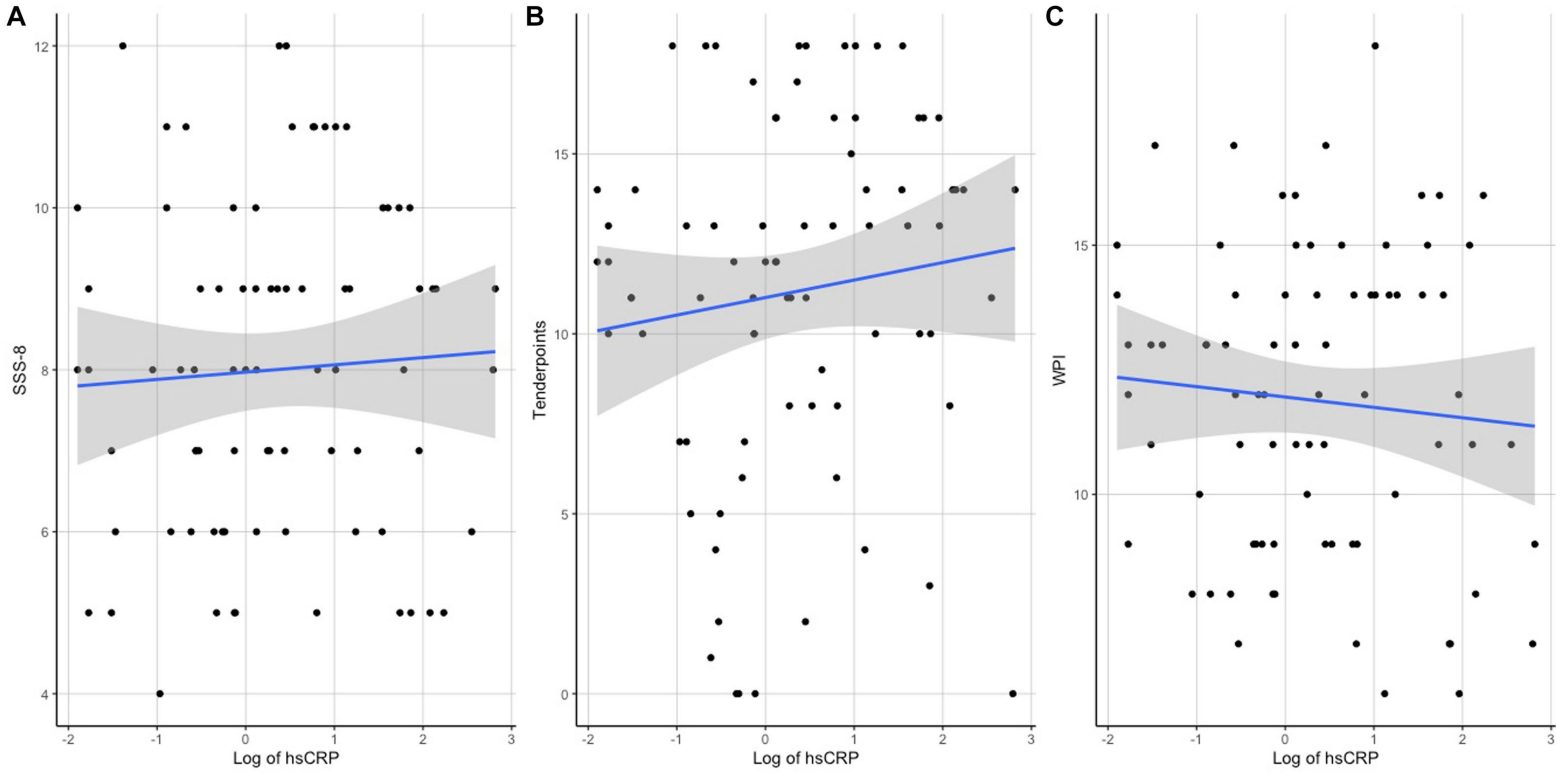
Scatterplots of hsCRP with clinical outcomes **(A)** SSS-8, **(B)** Tender Points, and **(C)** WPI.

**Figure 2:**
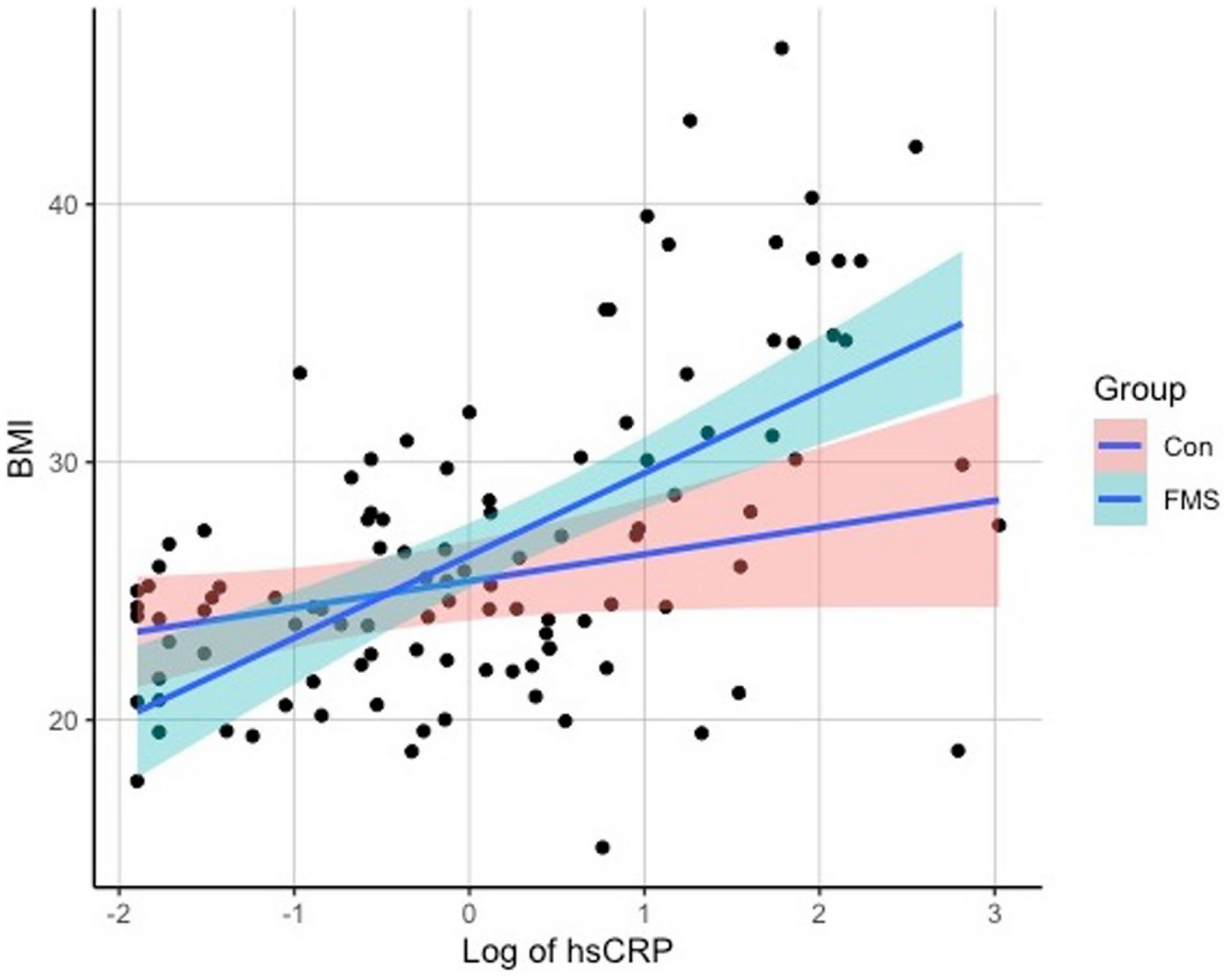
Scatterplot of hsCRP and BMI.

Regarding the associations of hsCRP with clinical measures, a correlation analysis revealed significant associations of hsCRP levels with the variables BMI (*r* = 0.465, *p* < 0.001), SSS-8 (*r* = 0.287, *p* = 0.008), and tender points (*r* = 0.307, *p* = 0.005), but not with WPI (*r* = 0.202, *p* = 0.073) or age (*r* = 0.06, *p* = 0.516). Scatterplots of the correlation analyses can be seen in Figure 1 for SSS-8, Tender Points, and WPI and Figure 2 for BMI.

**Figure 3:**
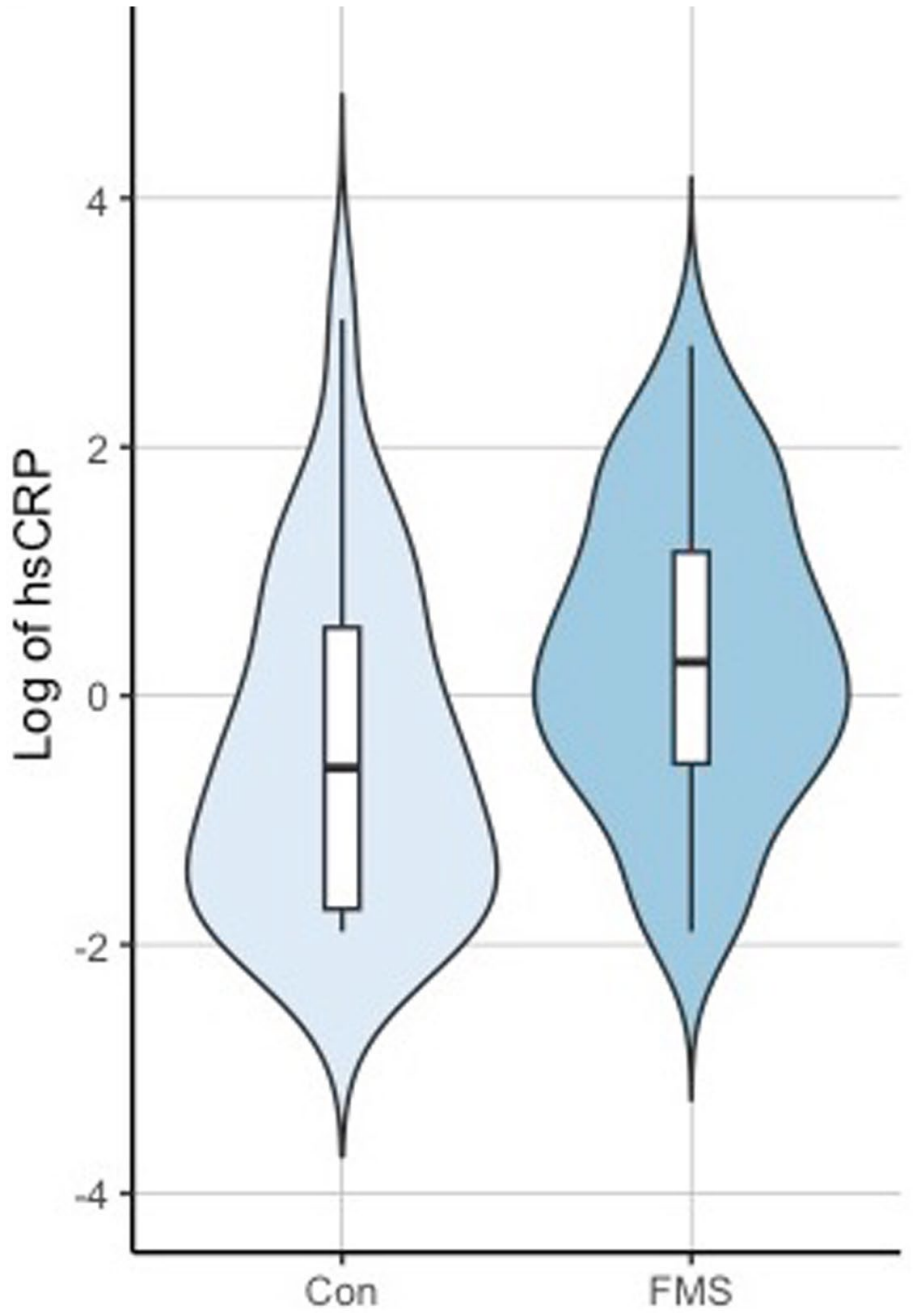
Violin plots of hsCRP Controls (Con) vs. Fibromyalgia (FMS).

To analyze whether the associations were driven by covariates, an ANCOVA was performed with the covariates overweight, age, smoking status, and gender. The results indicated a significant association between hsCRP levels and weight [*F*(1,102) = 20.815, *p* < 0.001, partial η^2^ = 0.1543] for overweight individuals. However, there was no significant association observed between hsCRP levels and age [*F*(1,102) =0.663, *p* = 0.42, partial η^2^ = 0.005], smoking status [*F*(1,102) = 0.2422, *p* = 0.623, partial η^2^ = 0.0018], or gender [*F*(1,102) = 0.601, *p* = 0.437, partial η^2^ = 0.0045], indicating that hsCRP levels are independent of age, smoking status, and gender. After adjusting for these covariates, the group effect (FMS vs. Controls) did not reach the significance threshold anymore [*F*(1,102) = 3.96, *p* = 0.052, partial η^2^ = 0.029]. Results on hsCRP differences between the groups are shown in Figure 3.

An estimated effect of +0.6 (95% credibility set [0.01,1.8]) was calculated for the group factor, +1.05 (95% credibility set [0.59,1.5]) for overweight, −0.01 (95% credibility set [−0.02, 0.01]) for age, −0.05 (95% credibility set [−0.31, 0.21]) for smoking, and 0.26 (95% credibility set [−0.34,0.86]) for gender, which is consistent with the findings of the ANCOVA analysis, indicating significant higher values in the FMS group. Prior to the analysis, a verification of the assumption for the ANCOVA model was fulfilled by testing for co-linearity and heteroscedasticity with a Levene test. The ANCOVA combined with a *post hoc* test (Turkey test), led to a statistically significant difference between the groups. Further, a controlled correlation analysis was performed to control for the variable weight, age, smoking, and gender. The partial correlation coefficient confirmed a significant correlation between hsCRP with SSS-8 (*r* = 0.216, *p* = 0.028), but not with tender points (*r* = 0.089, *p* = 0.37) and WPI (*r* = 0.011, *p* = 0.908).

## 4 Discussion

In this study, we investigated on serum concentrations of high-sensitivity C-reactive protein (hsCRP) in individuals with fibromyalgia (FMS) compared to pain-free controls. By addressing multiple dimensions related to hsCRP in the context of FMS, the research yielded noteworthy outcomes.

### 4.1 Elevated hsCRP concentrations in individuals with FMS

Our initial analysis showed that individuals with FMS had significantly higher hsCRP concentrations compared to pain-free controls, suggesting the possibility of increased systemic inflammation in individuals with FMS. To date, the data have been largely contradictory (8). Many earlier studies used the less sensitive CRP assays that were prevalent at the time, which did not capture subclinical inflammatory processes. In addition, another major weakness of previous studies was that they often did not ensure that participants with coexisting comorbidities (16–20, 33) or coexisting anti-inflammatory medication were excluded (11, 16–21, 33) or the diagnostic criteria were not clearly defined (8). Furthermore, previous literature focused on the ACR 1990 criteria and studies on hsCRP in the context of the revised diagnostic criteria were lacking (8). Our study shows that even with strict inclusion and exclusion criteria, evidence of elevated hsCRP in the subclinical range can be identified, confirming the association of FMS with subclinical inflammatory processes. However, after adjustment for confounding variables such as age, smoking status, and gender, the observed differences in hsCRP concentrations between the FMS and control group became statistically insignificant. This new observation suggests that hsCRP may not be directly related to FMS, but may rather reflect group differences in age, lifestyle, and especially gender. It is worth noting the significant gender differences between the groups in our study. Although a direct association between gender and hsCRP has not been previously described, our findings highlight the need for further research in this area. The mere presence of elevated hsCRP levels compared to pain-free controls does not conclusively establish causality. This calls for further investigation into the interplay between elevated CRP levels and clinical symptoms, and the potential influence of factors such as gender on these levels. Future studies are essential to clarify these relationships. The finding of increased hsCRP levels compared to pain-free controls does not yet allow a statement about causality. This raises the question of the relationship between elevated CRP levels and clinical symptoms.

### 4.2 Association between hsCRP concentrations and clinical measures

We further explored the relationship between hsCRP concentrations and clinical measures in FMS. Notably, we observed a significant association between higher symptom burden or a greater number of positive tender points and increased levels of hsCRP concentrations. This suggests that hsCRP, as an indicator of subclinical inflammatory processes, may serve as a potential biomarker reflecting disease severity in FMS. However, different roles of hsCRP need to be considered. On the one hand, as an expression of inflammatory processes, hsCRP may be a biomarker or even a causal factor in the development of FMS-associated complaints. This is supported by a large body of evidence showing a close interaction between inflammatory processes and processes of pain sensitization as well as psycho-cognitive complaints (34). At the same time, the literature agrees that anti-inflammatory drugs are of little or no importance in the treatment of FMS symptoms. This suggests that hsCRP may only have a moderating function and may be less causally involved in the pathogenesis of FMS than an expression of possible co-morbidities associated with both FMS and inflammatory processes. A central co-factor that is repeatedly discussed here is obesity, which is associated with both FMS and subclinical inflammatory processes (10, 35).

### 4.3 Correlation between hsCRP and BMI

Interestingly, we discovered a significant positive correlation between hsCRP levels and body mass index (BMI) in individuals with FMS. However, no significant correlations were found with age or smoking status. These findings are consistent with previous studies and lead to the assumption that weight has an influence on hsCRP levels (18, 23, 36). It has further been found, that FMS symptoms are related to BMI, with participants with higher BMI having a higher burden of pain and a higher severity of FMS associated symptoms (37, 38). The link between pain and obesity has been the subject of much debate. However, it is not yet clear how these factors interact. Cleary, several mediating factors play an important role in the interaction between pain and obesity. One example are pro-inflammatory markers as such as hsCRP. Pro-inflammatory markers represent inflammatory processes of the body and seem to contribute to pain symptoms as for example central pain sensitization (10). In line with this, obesity has been discussed as a process of low-grade inflammation in the body, which may be represented by markers of inflammation, such as hsCRP, for example, and overweight has been observed to be associated with higher pain sensitivity or widespread pain syndromes (36, 38, 39). The distinctive association between BMI and hsCRP levels in the FMS cohort, is a pattern that cannot be verified in the control group (see Figure 2). This is an interesting finding and suggests that while increasing BMI may be a factor to consider in health contexts generally, its interaction with inflammatory processes may be particularly relevant for those with FMS. A plausible interpretation of this phenomenon is that not all individuals respond in the same way to obesity-induced inflammatory processes. For certain individuals, perhaps predisposed by genetic, epigenetic, or other unidentified factors, an increase in BMI may act as a catalyst, triggering a clinical cascade with multiple effects. This cascade may manifest as an increased inflammatory response and potentially exacerbate fibromyalgia-related symptoms. Conversely, there appear to be individuals who, despite having an elevated BMI, do not exhibit the same inflammatory responses. This observed imbalance is consistent with the concept of “obesity resilience,” a phenomenon observed in relation to conditions such as non-alcoholic fatty liver disease (NAFLD) or diabetes (40, 41). These “resilient” individuals appear to tolerate higher BMIs without succumbing to the adverse effects typically associated with obesity. Despite its clear relevance to FMS, the literature on obesity resilience in the context of FMS remains sparse. In the future, it will be essential to extend our understanding beyond the simple cataloging of risk factors. A comprehensive approach should also seek to uncover protective mechanisms and factors that inhibit the progression from increased BMI to exacerbated FMS symptoms. Identifying and understanding these protective elements may open avenues for novel therapeutic strategies and interventions aimed at building resilience to the negative effects of obesity in FMS. Further, the relationship between pro-inflammatory markers, pain and obesity are not completely understood, but an interaction can clearly be described. Though, further factors must be considered in the relation and interplay of pain, obesity, and pro-inflammatory markers. HsCRP can therefore be used as clinical diagnostic factor, indicating inflammatory processes and higher severity of FMS associated symptoms. Consequently, weight reduction could play a central role in the reduction of inflammation and pain and has been found to reduce CRP concentrations and may therefore be a crucial parameter for the therapy of patients with FMS and the reduction of pain (12, 37).

The conclusion of this study is that sub-inflammatory processes are associated with the clinical symptoms of fibromyalgia syndrome. Although some of the association can be attributed to differences in weight, the link between sub-inflammatory processes and FMS cannot be fully explained by this factor. Previous attempts to treat the symptoms of FMS with anti-inflammatory therapies have not been successful (42, 43), so the exact clinical significance of the evidence of subclinical inflammation remains unclear. This highlights the need for further research to better understand the mechanisms underlying the association between hsCRP and FMS, and to explore the potential use of targeting hsCRP in the management of FMS. This study’s findings offer a starting point for future research that could have significant implications for the treatment and management of this complex and debilitating condition.

## Data availability statement

The raw data supporting the conclusions of this article will be made available by the authors, without undue reservation.

## Ethics statement

The studies involving humans were approved by Medical Faculty of the University of Heidelberg Ethics Committee (S-923/2019). The studies were conducted in accordance with the local legislation and institutional requirements. The participants provided their written informed consent to participate in this study.

## Author contributions

EB, SB, H-CF, and JT contributed to the conception and design of the study. EB and SB organized the database. SB performed the statistical analysis. EB wrote the first draft of the manuscript. SB and JT wrote the sections of the manuscript. All authors contributed to the article and approved the submitted version.

## Member of PerPAIN consortium

Andrei Sirazitdinov, Anita Schick, Annette Löffler, Christian Ruckes, Eva Beiner, Herta Flor, Jonas Tesarz, Jürgen Hesser, Leonie Ader, Martin Löffler, Michael Hopp, Stephanie Vock, Ulrich eininghaus, Wolfgang Eich.
